# Adaptive responses of neuronal cells to chronic endoplasmic reticulum (ER) stress

**DOI:** 10.1016/j.redox.2023.102943

**Published:** 2023-10-20

**Authors:** Thu Nguyen Minh Pham, Natarajan Perumal, Caroline Manicam, Marion Basoglu, Stefan Eimer, Dominik C. Fuhrmann, Claus U. Pietrzik, Albrecht M. Clement, Hagen Körschgen, Jana Schepers, Christian Behl

**Affiliations:** a Institute of Pathobiochemistry, University Medical Center of the Johannes Gutenberg University, Mainz, Germany; b Department of Ophthalmology, University Medical Center of the Johannes Gutenberg University, Mainz, Germany; c Department of Structural Cell Biology, Institute for Cell Biology and Neuroscience, Goethe University, Frankfurt am Main, Germany; d Institute of Biochemistry I, Faculty of Medicine, Goethe-University Frankfurt, Frankfurt, Germany

**Keywords:** ER stress resistance, ER-Phagy, Giant lysosomes, Aerobic glycolysis, Warburg effect, Neuroprotection

## Abstract

Accumulation of misfolded proteins or perturbation of calcium homeostasis leads to endoplasmic reticulum (ER) stress and is linked to the pathogenesis of neurodegenerative diseases. Hence, understanding the ability of neuronal cells to cope with chronic ER stress is of fundamental interest. Interestingly, several brain areas uphold functions that enable them to resist challenges associated with neurodegeneration. Here, we established novel clonal mouse hippocampal (HT22) cell lines that are resistant to prolonged (chronic) ER stress induced by thapsigargin (TgR) or tunicamycin (TmR) as *in vitro* models to study the adaption to ER stress. Morphologically, we observed a significant increase in vesicular und autophagosomal structures in both resistant lines and ‘giant lysosomes’, especially striking in TgR cells. While autophagic activity increased under ER stress, lysosomal function appeared slightly impaired; in both cell lines, we observed enhanced ER-phagy. However, proteomic analyses revealed that various protein clusters and signaling pathways were differentially regulated in TgR versus TmR cells in response to chronic ER stress. Additionally, bioenergetic analyses in both resistant cell lines showed a shift toward aerobic glycolysis (‘Warburg effect’) and a defective complex I of the oxidative phosphorylation (OXPHOS) machinery. Furthermore, ER stress-resistant cells differentially activated the unfolded protein response (UPR) comprising IRE1α and ATF6 pathways. These findings display the wide portfolio of adaptive responses of neuronal cells to chronic ER stress. ER stress-resistant neuronal cells could be the basis to uncover molecular modulators of adaptation, resistance, and neuroprotection as potential pharmacological targets for preventing neurodegeneration.

## Highlights

• Chronic ER stress induces autophagic vesicles and ‘giant’ lysosomes in neuronal cells.
• Cells show enhanced levels of ER-phagy and increased levels of RTN3L.
• Both cell lines show a metabolic shift toward aerobic glycolysis (Warburg's effect).
• The IRE1 and ATF6 pathways of the UPR are activated in ER-stress adapted cells.

## Graphical abstract

**Figure:**
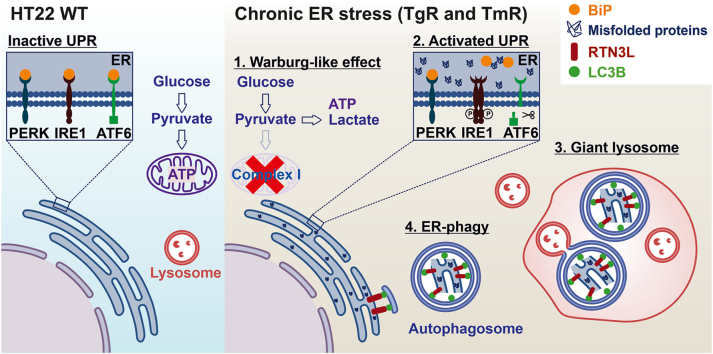

## 1 Introduction

The endoplasmic reticulum (ER) as the largest organelle in the cell is a major hub for protein quality control processes and intracellular signaling. It consists of the nuclear envelope, sheet-like cisternae, and polygonal tubules with three-way junctions that are classified by their membrane structure [1]. Ribosome-decorated ER sheets play a crucial role in the synthesis, folding, and modification of proteins. Ribosome-free regions of transitional tubular ER engage in lipid and sterol biosynthesis, inter-organelle contacts, and serve as a Ca^2+^ reservoir [1].

Disruption of ER structure and function results in the accumulation of mis- or unfolded proteins in the luminal ER, referred to as “ER stress” [2]. As an adaptive mechanism to reinstate ER homeostasis and ensure correct protein folding, cells have evolved the unfolded protein response (UPR). During ER stress, the binding immunoglobulin protein (BiP/GRP78/HSPA5) preferentially binds to unfolded/misfolded proteins, thereby releasing and activating three UPR transmembrane proteins, the protein kinase RNA-activated-like ER kinase (PERK), the inositol requiring protein 1α (IRE1α), and the activating transcription factor 6 (ATF6) [3]. Activated PERK phosphorylates the α subunit of eukaryotic initiation factor 2 (eIF2α), transiently suppressing global translation, and concomitantly promoting activating transcription factor 4 (ATF4) activity to strengthen the antioxidant machinery, folding capacity, and induce protein clearance by autophagy. Upon ER stress, RNase activity of IRE1 cleaves a 26 base pairs-long intron of X-Box binding protein 1 (*Xbp1*) mRNA. *Xbp1s* (Xbp1's spliced form) is an active transcription factor that modulates gene expression of factors involved in protein translocation into the ER, folding, and secretion, and degradation of misfolded proteins. After release from BiP, full-length ATF6 undergoes proteolysis at the Golgi apparatus. ATF6 fragments transcriptionally regulate ER chaperones and enzymes for ER protein translocation, folding, maturation, and secretion, as well as the degradation of misfolded proteins [4]. Chronic ER stress and persistent UPR activation cause cell death.

Aberrant ER-luminal proteins are translocated to the cytoplasm by the ER-associated protein degradation (ERAD) machinery. Subsequently, the proteins are degraded by the ubiquitin-proteasome system (UPS) [5] or, alternatively, by autophagy (“self-eating”) [6]. Macroautophagy (hereafter referred to as autophagy) is an evolutionarily conserved process that sequesters damaged or non-functional components (e.g., proteins or organelles) in double-membraned vesicles (autophagosomes), which eventually fuse with the lysosome for degradation [6]. Autophagic degradation plays a key part in maintaining proteostasis under physiological and stress conditions and can be highly selective. Misfolded proteins are cleared by a selective autophagy pathway that relies on the involvement of autophagy receptors providing cargo selectivity [7]. The selective autophagy pathway that clears ER-fragments, ER-phagy, is induced by ER stress as well as starvation, ribosome stalling, bacterial infection, and accumulation of polypeptides [8].

Disturbed proteostasis and ER stress are key pathogenetic processes in neurodegeneration [[9], [10], [11]]. In Alzheimer's disease (AD), for instance, aberrant proteostasis [[12], [13], [14]], altered metabolic homeostasis, and enhanced oxidative stress [15,16], together with neuroinflammation, microgliosis, and astrogliosis [17] are found. Postmortem brains of AD patients, as well as animal models, display increased ER stress and UPR activation [[18], [19], [20]].

In neurodegeneration, many brain areas remain functional by adapting to the triggers of degeneration, as shown by an upregulated antioxidative response in AD brains and *in vitro* [[21], [22], [23], [24]]. Based on previous cell studies showing successful adaptation to chronic oxidative stress [[22], [23], [24], [25], [26], [27]], we generated novel clonal mouse hippocampal (HT22) cell lines that substantially resist chronic ER stress induced by thapsigargin (Tg) or tunicamycin (Tm). While Tg acts as a non-competitive inhibitor of the sarco/endoplasmic reticulum (ER) Ca^2+^-ATPase (SERCA) [28], Tm blocks protein N-glycosylation [29]. Generated resistant cell lines were named TgR and TmR.

Here, we show that chronic treatment of neuronal cells with ER stressors caused changes in intracellular vesicles and an increased autophagic flux. Further, a wide portfolio of protein expression patterns related to stress adaptation and signaling pathways including ER-phagy and the UPR were altered. Intriguingly, we detected significantly altered ATP production in ER stress-resistant cells and a metabolic shift to aerobic glycolysis (‘Warburg effect’). Taken together, this work uncovered pathways of adaptability in neuronal cells to chronic ER stress. Interestingly, we observed a possible mechanistic link between ER stress, altered metabolisms, and autophagic-lysosomal function.

## 2 Materials and methods

### 2.1 Chemicals and antibodies

**Table:** 

Resources	Source	Catolog number	
Chemicals			
Dimethylsulfoxide (DMSO)	Carl Roth	A994.1	
Thapsigargin	Sigma	T9033	
Tunicamycin	Sigma	T7756	
Bafilomycin A1	Biozol	TRCB110000	
Doxycycline	Sigma	D9891	
DMEM	Thermo Fisher Scientific	41965039	DMEM
EBSS	Thermo Fisher Scientific	24010043	
Glucose-free DMEM	Thermo Fisher Scientific	11966025	Glucose-free DMEM
Fetal bovine serum (FBS)	Thermo Fisher Scientific	10437028	Fetal bovine serum (FBS)
Sodium pyruvate	Gibco	11360070	Sodium pyruvate
Penicillin/streptomycin	Invitrogen	15140122	Penicillin/streptomycin
DPBS	Invitrogen	14190169	DPBS
EDTA-free protease inhibitor	Roche	11873580001	EDTA-free protease inhibitor
PhosSTOP™	Roche	4906845001	PhosSTOP™
Sodium chloride	Sigma	S7653	Sodium chloride
HEPES	Sigma	H3375	HEPES
Disodium hydrogen phosphate dihydrate	Carl Roth	4984	Disodium hydrogen phosphate dihydrate
Calcium chloride	Carl Roth	1C34.4	Calcium chloride
3-(4,5-dimethylthiazol-2-yl)-2,5-diphenyltetrazoliumbromide (MTT)	Sigma	M5655	3-(4,5-dimethylthiazol-2-yl)-2,5-diphenyltetrazoliumbromide (MTT)
Oligomycin	Cayman Chemical	11342	Oligomycin
carbonyl cyanide *m*-chlorophenylhydrazone (FCCP)	Cayman Chemical	15218	carbonyl cyanide *m*-chlorophenylhydrazone (FCCP)
Rotenone	Cayman Chemical	13995	Rotenone
Antimycin A	Sigma	A8647	Antimycin A
LysoTracker Red DND-99	Thermo Fisher Scientific	L7528	LysoTracker Red DND-99
Paraformaldehyde	Sigma	P6148	Paraformaldehyde
BSA	Sigma	A2153	BSA
PROTEOSTAT	Enzo Life Sciences	ENZ-51035-0025	PROTEOSTAT
DAPI	Calbiochem	382061	DAPI
Tris-HCl	Carl Roth	9090.3	Tris-HCl
EDTA	Fluka	0369	EDTA
EGTA	Carl Roth	3054	EGTA
Dithiothreitol	Sigma	D9779	Dithiothreitol
Triton X-100	Sigma	T9284	Triton X-100
Cathepsin D/E substrate	Enzo Life Sciences	BML-P145	Cathepsin D/E substrate
**Antibodies**			
LAMP2	DSHB Biology	ABL-93	LAMP2
CTSD	Abcam	ab75852	CTSD
HSP90	Stressgen	SPA-830	HSP90
LC3B	Sigma	L7543	LC3B
p62	Progen	GP62-C	p62
Tubulin	Sigma	T9026	Tubulin
RTN3	Proteintech	12055-2-AP	RTN3
RFP	Invitrogen	R10367	RFP
OXPHOS	Abcam	ab110413	OXPHOS
phospho-AMPKα (Thr172)	Cell Signaling	2535	phospho-AMPKα (Thr172)
AMPK	Cell Signaling	2532	AMPK
GRP78	BD Transduction Laboratories™	610978	GRP78
Actin	Sigma	A5060	Actin
IRE1α	Thermo Fisher Scientific	MA5-14991	IRE1α
phospho-eIF2α (Ser51)	Cell Signaling	9721	phospho-eIF2α (Ser51)
eIF2α	Cell Signaling	9722	eIF2α
ATF6	Novus Biologicals	NBP1-75478	ATF6
DLP1	BD Transduction Laboratories™	611113	DLP1
OPA1	BD Transduction Laboratories™	612607	OPA1
phospho-mTOR (Ser2448)	Cell Signaling	2971	phospho-mTOR (Ser2448)
mTOR	Cell Signaling	2972	mTOR
phospho-p70S6K (Thr389)	Cell Signaling	9206	phospho-p70S6K (Thr389)
p70S6K	Cell Signaling	9202	p70S6K
phospho-ULK1 (Ser555)	Cell Signaling	5869	phospho-ULK1 (Ser555)
ULK1	Cell Signaling	8054	ULK1
BECN1	Cell Signaling	3495	BECN1
ATL3	Proteintech	16921-1-AP	ATL3
FAM134A	Thermo Fisher Scientific	PA5-69633	FAM134A
FAM134B	Proteintech	21537-1-AP	FAM134B
FAM134C	Sigma	HPA016492	FAM134C
TEX264	Proteintech	25858-1-AP	TEX264
CCPG1	Proteintech	13861-1-AP	CCPG1
SEC62	Invitrogen	PA5-53119	SEC62
Peroxidase AffiniPure Donkey Anti-Mouse IgG (H + L)	Jackson ImmunoResearch	715-035-151	Peroxidase AffiniPure Donkey Anti-Mouse IgG (H + L)
Peroxidase AffiniPure Donkey Anti-Rabbit IgG (H + L)	Jackson ImmunoResearch	711-035-152	Peroxidase AffiniPure Donkey Anti-Rabbit IgG (H + L)
Peroxidase AffiniPure Donkey Anti-Guinea Pig IgG (H + L)	Jackson ImmunoResearch	706-035-148	Peroxidase AffiniPure Donkey Anti-Guinea Pig IgG (H + L)
Cy™2 AffiniPure Donkey Anti-Rat IgG (H + L)	Jackson ImmunoResearch	712-225-153	Cy™2 AffiniPure Donkey Anti-Rat IgG (H + L)
Alexa Fluor® 488 AffiniPure Donkey Anti-Rabbit IgG (H + L)	Jackson ImmunoResearch	711-545-152	Alexa Fluor® 488 AffiniPure Donkey Anti-Rabbit IgG (H + L)
Alexa Fluor® 647 AffiniPure Donkey Anti-Rabbit IgG (H + L)	Jackson ImmunoResearch	711-605-152	Alexa Fluor® 647 AffiniPure Donkey Anti-Rabbit IgG (H + L)

### 2.2 Cell culture

Thapsigargin- or Tunicamycin-resistant cell lines (HT22 TgR or TmR, respectively) were established from wildtype HT22 mouse hippocampal neuronal cell line (HT22 WT) by clonal selection adapted from the procedure of oxidative stress-resistant HT22 cells [27]. The HT22 WT cells that survived for 48 h in a sublethal concentration of Tg or Tm were subsequently cultured for three passages in the presence of ER stressors before increasing their concentration. The final desired concentration of Tg or Tm would be lethal for HT22 WT but not for TgR or TmR.

All cell lines were cultivated in Dulbecco's modified Eagle's medium (DMEM) containing 10 % fetal bovine serum (FBS), 1 mM sodium pyruvate, and 1x penicillin/streptomycin, supplemented with either 1.5 μM Tg or 1.5 μg/ml Tm. The ER stressors were omitted from the medium three days before cells were used for experiments to minimize an acute effect of the toxins.

For some experiments, cells were transiently transfected by calcium phosphate precipitation. Briefly, DNA-calcium phosphate precipitate was generated by adding a buffered saline/phosphate solution (NaCl 280 mM, HEPES 50 mM, Na_2_HPO_4_ 1.5 mM) to a mixture of DNA with CaCl_2_ 2 M in a dropwise manner, and subsequently incubated at room temperature for 30 min. The precipitate was added to the cells for further assays.

### 2.3 Cell viability assay

Cell viability was assessed by cellular metabolic activity via the capacity of cells for reducing 3-(4,5-dimethylthiazol-2-yl)-2,5-diphenyltetrazoliumbromide (MTT) to formazan, blue-violet colored crystals. They were determined colorimetrically over desired time courses. The procedure has been reported previously [30].

### 2.4 Transmission electron microscopy

Cells were washed three times with PBS and then pre-fixed with 2.5 % glutaraldehyde in 0.1 M cacodylate buffer pH 7.2 for 2 h at room temperature. After two washes in 0.1 M cacodylate buffer containing 2 % sucrose, cells were post-fixed in 1 % reduced osmium tetroxide, dehydrated, and embedded in Araldite resin. 50-nm sections were cut with an ultramicrotome (Leica). Ribbons of sections were transferred on Formvar-coated copper slot grids and enhanced contrast with 5 % uranyl acetate in methanol/water and lead citrate [31]. Micrographs were taken with a Zeiss TEM900 microscope operated at 80 keV in the bright-field mode and quipped with a Troendle 2K camera.

### 2.5 ER-phagy assay

ER-phagic flux was determined as previously reported [32]. Briefly, the doxycycline-inducible ER-phagy reporter (ssRFP-GFP-KDEL) was transiently expressed in HT22 WT and both ER stress-resistant cell lines using DNA-calcium phosphate precipitation. Upon ER-phagy, lysosomal hydrolases cause degradation of the linker between GFP and RFP and release stable GFP-free RFP fragments, which are then detected in either fluorescence microscopy or immunoblotting.

### 2.6 Quantitative real time PCR array

RNA extraction, cDNA synthesis, and quantitative real‐time PCR (qPCR) for HT22 WT, TgR, and TmR cells were based on the conceptual framework proposed by Hiebel et al. [33]. A primer library (Biomol, MATPL-1) including 88 primer sets directed against autophagy genes was performed. PCR protocols were performed according to the manufacturer's instructions. Relative expression was calculated and normalized to *Gapdh* using the REST software [34]. Fold changes of greater than 1.5 or lower than 0.6 were considered as a significant up- or down-regulation, respectively.

Primers used for qPCR were as follows: *Xbp1s* GAGTCCGCAGCAGGTG (forward), GTGTCAGAGTCCATGGGA (reverse); *Atf4* GCATGCTCTGTTTCGAATGGA (forward), CCAACGTGGTCAAGAGCTCAT (reverse); *Ddit3* CACATCCCAAAGCCCTCGCTCTC (forward), TCATGCTTGGTGCAGGCTGACCAT (reverse).

### 2.7 Proteomics analysis

Label-free quantitative discovery proteomics analysis and the corresponding steps such as sample preparation, protein extraction, nano-liquid chromatography–electrospray ionization–MS/MS (nLC-ESI-MS/MS) analysis, and bioinformatics analyses to elucidate the distinct functional annotation and pathways employing the Ingenuity Pathway Analysis (IPA) tool were carried out according to the procedures described elsewhere [35,36]. Detailed parameters for this analysis are described in Supplementary Data S6.

### 2.8 Live metabolic flux assays

The cellular oxygen consumption rate (OCR) and the extracellular acidification rate (ECAR) were analyzed using a Seahorse XFe96 extracellular flux analyzer (Agilent). 10,000 cells/well were seeded in Seahorse 96-well cell culture plates. On the day of measurement, cells were equilibrated 1 h before measurement in XF DMEM medium supplemented with 10 mM l-glucose, 2 mM l-glutamine, and 1 mM pyruvate at 37 °C and ambient CO_2_. For Mito Stress Test, cells were treated with 2.5 μM oligomycin, a complex V inhibitor to block ATP-coupled respiration, 1 μM FCCP to uncouple the respiratory chain, and 500 nM rotenone together with 1 μg/ml antimycin A, complex I and III inhibitor respectively, to block mitochondrial respiration. To analyze the ATP production rate, 2.5 μM oligomycin and 500 nM Rotenone together with antimycin A were sequentially added to the cells. Data were processed using Wave Desktop (Version 2.6.0.31) and ATP rates were calculated using the Seahorse Analytics online tool (Version 1.0.0–570).

### 2.9 Nicotinamide adenine dinucleotide (NAD+/NADH) assay

The ratio of NAD^+^/NADH was determined by fluorometric assay kit (ab176723, Abcam). Briefly, 25 μl of NAD/NADH control buffer or NADH extraction buffer was added to lysate samples, and then, incubated at 37 °C for 10 min. Another 25 μl of the buffer control or NAD extraction buffer, respectively, was added to neutralize the extractions. Next, 75 μl of NAD/NADH reaction mixture was added into each wells, subsequently incubated at room temperature for 60 min in the darkroom. Fluorescence signals were measured at ex/em 540/590 nm.

### 2.10 Immunoblotting and immunocytochemistry

Immunoblot analyses were accomplished as described previously [37]. Briefly, 20 μg of total protein was subjected to SDS-PAGE on Bis-Tris gels. After transferring the proteins on nitrocellulose membrane, blots were developed with specific antibodies (1:1000) (see Chemicals and antibodies) and enhanced chemiluminescence signals were detected with the Amersham™ Imager 600 (GE Healthcare Europe, Freiburg, Germany). Quantification was carried out using Aida Image Analyzer v4.26 software (Raytest, Straubenhardt, Germany).

The autophagic flux was calculated by subtracting normalized LC3B-II protein levels of untreated samples from the corresponding samples treated with bafiA1.

For immunocytochemistry, cells were plated on glass coverslips in 24-well plates. For the labelling of lysosomes and acidic vacuoles, 1 μM LysoTracker Red DND-99 was added in complete medium for 10 min under culture conditions. Then, the cells were fixed with 4 % (w/v) paraformaldehyde for 15 min and treated with ice-cold 90 % methanol for 6 min. Nonspecific binding sites were blocked with 5 % (w/v) BSA in PBS before incubating with the primary antibody (1:200 in 1 % [w/v] BSA). Subsequently, cells were incubated with a fluorophore-conjugated secondary antibody Cy2, Alexa Fluor™ 488, or Alexa Fluor™ 647 (1:200 in PBS), and DAPI. When applicable, PROTEOSTAT dye (1:500) was incubated with DAPI for 30 min at room temperature. Confocal micrographs were acquired with the laser-scanning microscope LSM 710 (Zeiss).

### 2.11 Lysosomal fractionation and cathepsin D/E activity assay

Cells were scraped in cold PBS and lysed in a hypotonic lysis buffer (20 mM Tris-HCl, 1 mM EDTA, 1 mM EGTA, 1 % glycerol, 2 mM dithiothreitol, pH 7.8), then centrifuged at 13,000 rpm for 30 min at 4 °C. The lysosomal membrane was disrupted using a lysis buffer (200 mM sodium acetate, 50 mM NaCl, 0.1 % Triton X-100, pH 5.0) with sonication (amplitude 60 %, two consecutive 10 s intervals), followed by another centrifugation at 13,000 rpm for 30 min at 4 °C. Protein concentration was determined using bicinchoninic acid protein assay kit (Thermo Scientific).

CTSD activity was determined by measuring the release of fluorescent aminomethyl coumarin (AMC)-containing peptide as previously described [38]. One microgram of lysosomal protein in 50 μl assay buffer on a black 96-well plate was incubated at 37 °C for 15 min. Subsequently, 50 μl of 20 μM cathepsin D/E substrate was supplemented in a total volume of 100 μl with or without pepstatin A to each sample. The plate was incubated at 37 °C for 60 min and fluorescent intensity was measured every 30 s (ex/em 340/420 nm) with a Varioscan Lux (Thermo Fisher).

### 2.12 Statistical analysis

Statistical differences of all quantified immunoblots and cell viability assay were determined by ordinary one-way ANOVA or Student's t-test as appropriate; differences between treatments were determined by Bonferroni's post hoc test. Seahorse measurement assays were analyzed by two-way ANOVA using GraphPad Prism 7 (GraphPad Inc.). p < 0.05 was considered statistically significant. The results are expressed as mean ± S.E.M. The proteomics data were subjected to Student's two-sided *t*-test statistical analysis using the Perseus software (version 1.6.1.0) to identify significantly differentially abundant proteins.

## 3 Results

### 3.1 TgR and TmR cells show an increased number of autophagic vesicles

TgR and TmR clonal hippocampal HT22 cells were established by continuous exposure to increasing concentrations of Tg or Tm and selection of surviving cells. Growth rates of TgR and TmR cells were lower than those of HT22 wild type (WT) controls (Fig. 1A and B), indicating that ER stress resistance is not associated with increased cell proliferation; several selected subclones of TgR and TmR behaved similarly (data not shown). TgR and TmR cells did not exhibit cross-resistance (Fig. S1A), suggesting that resistance is specific to the individual challenge (calcium or glycosylation stress).

**Fig. 1:**
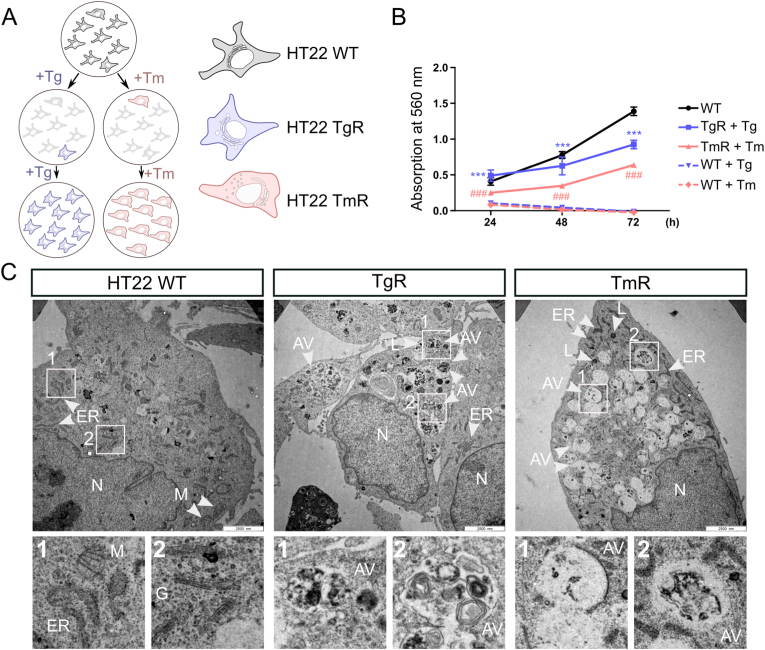
Generation procedure and ultrastructural analysis of ER stress-resistant HT22 cells A. Schematic representation of the selection process of TgR or TmR cells from HT22 WT cells. Parental HT22 cells were treated with desired concentrations of either thapsigargin (Tg) or tunicamycin (Tm). The survival clones were isolated and exposed to higher concentrations of the stressors. Eventually, the ER stress-resistant HT22 clones were resistant to 1.5 μM Tg or 1.5 μg/ml Tm, hereafter known as thapsigargin-resistant HT22 cells (TgR), tunicamycin-resistant HT22 cells (TmR), respectively. B. Proliferation rate of HT22 WT, TgR, and TmR cells with either Tg 1.5 μM or Tm 1.5 μg/ml was measured by MTT assay over 72 h. Values represent mean ± S.E.M., n = 4, ***p < 0.001 TgR + Tg group vs. WT + Tg group, ###p < 0.001 TmR + Tm vs. WT + Tm group. C. EM images of HT22 WT, TgR, and TmR cells under basal conditions. TgR and TmR cells showed an increased number and size of degradative compartments, which were wrapped in large blebs in TgR or definite vesicles in TmR cells. Autophagic vesicles (AV), lysosomes (L), endoplasmic reticulum (ER), mitochondria (M), Golgi apparatus (G), and nucleus (N). Scale bar, 2500 nm.

Our previous work on oxidative stress-resistant HT22 cells showed alterations in overall autophagic vesicles and mitochondrial morphology [27]. Employing transmission electron microscopy (TEM) in both, TgR and TmR, we observed an elevated number of autophagic vesicles (AV) (characterized by two limiting membranes), autophagic compartments containing incompletely digested substrates (amorphous electron-dense content), and dense (and partially very large) degradative lysosomes, indicating enhanced autophagic activity (Fig. 1C, Fig. S1B). Some of the enlarged compartments comprised AVs and formed larger ‘membrane blebs’ in TgR, while TmR cells displayed many distinct vesicular compartments. Further, we observed ER-mitochondria contact sites in WT cells (Fig. S1Ba-c) but not in resistant cells, suggesting decoupling of ER and mitochondria under chronic ER stress. Moreover, we observed expanded ER in TgR (Fig. S1Bd) and a number of ER fragments in TmR (Fig. S1Bg), indicating the impact of ER stress and ongoing ER degradation and turnover. Mitochondrial network morphology also displayed changes under ER-stress, which was more pronounced in TmR cells (Fig. 1C, Fig. S1B). Consistently, we detected an altered expression of mitochondrial fission marker dynamin-1-like protein (DLP1) in both resistant cell lines, and altered protein levels of mitochondrial fusion marker dynamin-like 120 kDa protein (OPA1) in TmR, underlining altered mitochondrial dynamics in ER stress adaptation and resistance (Fig. S1C).

### 3.2 ‘Giant lysosomes’ accumulate in ER stress-resistant cells

To verify that observed enlarged degradative compartments were indeed lysosomes, we employed a pH-sensitive fluorescent dye (DND-99 LysoTracker) together with antibodies against either lysosome membrane associated protein 2 (LAMP2) or aspartic protease cathepsin D (CTSD). LysoTracker is an acidotrophic probe that marks protonated lysosomes and prominently colocalized with both lysosomal markers in control cells. In a subpopulation of TgR and TmR, tracker and markers colocalized at giant vesicles, confirming that the large structures observed via TEM are indeed lysosomes. (Fig. 2A). Moreover, both resistant cells showed increased numbers of ‘giant lysosomes’. We then further examined lysosomal cathepsins. During transportation to lysosomes, inactive preproenzyme CTSD is cleaved and glycosylated to form an active single-chain intermediate located in endosomes. Final processing yields two mature subunits including heavy and light chains [39]. Fig. 2B displays higher levels of preproCTSD (46 kDa) and proCTSD (43 kDa) in TmR while the levels of the mature form (heavy chain) of CTSD remained unchanged. Moreover, using a CTSD/E-specific fluorogenic substrate, we detected a decreased CTSD/E activity in ER stress-resistant cells (Fig. 2C). Next, we employed anti-CTSD and PROTEOSTAT dye, a specific probe for misfolded and aggregated proteins. Overall, both resistant lines showed an increase in misfolded/aggregated proteins that were dense in CTSD-positive compartments (Fig. S2). This suggests an increased misfolded/aggregated protein load in potentially impaired lysosomes, resulting in ‘giant lysosomes’ in ER stress-resistant cells. Taken together, these results showed that chronic ER stress can significantly change lysosomal morphology and, potentially, also partly impair function.

**Fig. 2:**
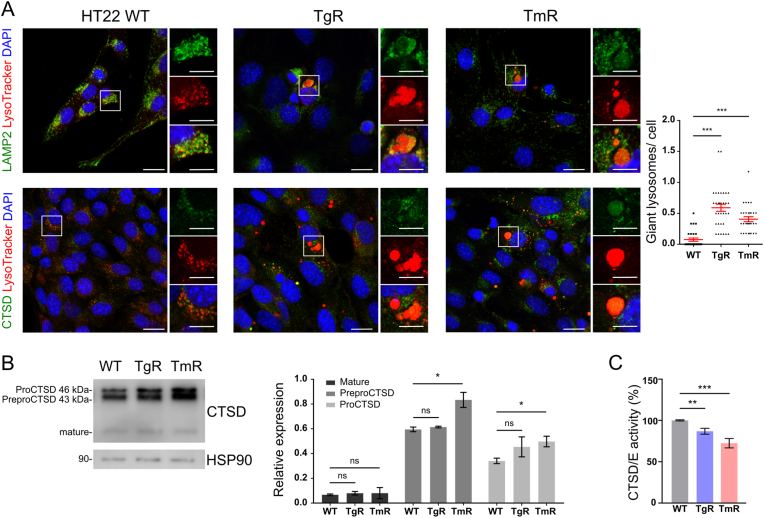
Impaired lysosomes in TgR and TmR cells A. Immunofluorescence staining showed that LysoTracker red-positive ‘giant vesicular’ compartments were colocalized with LAMP2 and cathepsin D (CTSD) in untreated HT22 WT, TgR and TmR cells. DAPI (blue) was used to stain DNA. Scale bars: 20 μm. Values represent mean ± S.E.M., n = 3 (150–250 cells per group), ∗∗∗p < 0.001. B. Western blotting analysis displayed increases in immature forms of CTSD in TmR cells compared with HT22 WT cells. HSP90 was used as loading control. Values represent mean ± S.E.M., n = 3, ns non-significant, *p < 0.05 compared with the corresponding groups of the WT cells C. Relative cathepsin D/E activity of ER stress-resistant cells was reduced compared to HT22 WT cells. Values represent mean ± S.E.M., n = 3, ∗∗p < 0.01, ∗∗∗p < 0.001. (For interpretation of the references to color in this figure legend, the reader is referred to the Web version of this article.)

### 3.3 Autophagic flux and ER-phagy are enhanced in TgR and TmR cells

The observed overall vesicular ultrastructure suggested an enhanced autophagic activity in TgR and TmR. Therefore, we monitored LC3-II turnover as a measure of autophagic flux in the presence and absence of bafiA1, an inhibitor of autophagosome-lysosome fusion. Indeed, TgR showed an increased LC3-II flux under basal conditions compared with WT cells, which further increased following EBSS-induced starvation (Fig. 3A). Interestingly, TmR showed no changes in baseline autophagic activity, but an increased autophagic response upon starvation (Fig. 3A). The observed differential autophagic flux indicates that while the stressors employed (Tg and Tm) are both ER stressors, they still provoke a different autophagic response. Phosphorylation of mammalian target of rapamycin (mTOR), key upstream negative regulator of canonical autophagy, was unchanged in both TgR and TmR (Fig. S3A). Phosphorylated Thr389 of serine/threonine kinase p70^S6K^, a downstream target of mTOR, was also unchanged (Fig. S3A). However, TgR cells exhibited an increased phosphorylation of serine/threonine kinase ULK1 (S555), one central regulator in autophagy initiation, consistent with the observed elevated autophagic flux (Fig. S3B). Next, we examined Beclin-1 (BECN1) expression. BECN1 is a regulatory subunit of the class III phosphatidylinositol 3-kinase complex I (PI3KC3–C1) needed for canonical membrane elongation; it is also related to the endocytic pathway and tumor-suppression [40,41]. Compared with WT cells, TmR showed an increased BECN1 expression (Fig. S3B), suggesting non-autophagic functions of BECN1 in response to chronic ER stress.

**Fig. 3:**
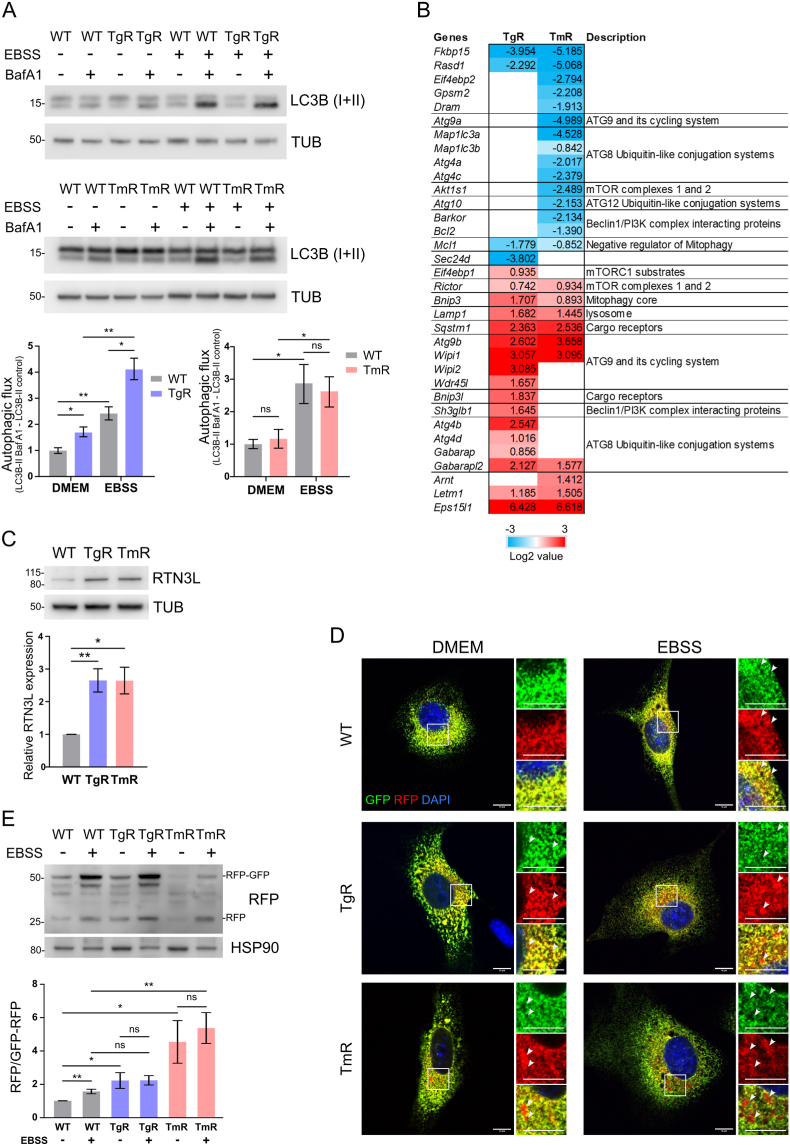
Induction of autophagy and ER-phagy in chronic ER stress A. Immunoblot analyses depicted autophagic activity under basal and nutrient-deprived conditions in the stress-resistant cells, TgR and TmR. Cells were starved for 2 h, then treated with DMSO (control) or 2 μM bafilomycin A1 (BafA1) for additional 4 h to evaluate LC3-II flux. LC3-II levels were normalized to the loading control Tubulin (TUB). Statistics are depicted as mean ± S.E.M. TgR: n = 3, TmR: n = 4, ns non-significant, *p < 0.05, **p < 0.01. B. Heatmap showed significant changes in autophagy-associated gene expression in ER stress-resistant cell lines compared to HT22 WT. Levels of mRNA were determined by RT-qPCR against the mouse Autophagy Primer Library 1 (MATPL-1). Relative fold change in gene expression was normalized to *Gapdh* mRNA level. Log2 values of +0.585 or −0.737 (>1.5-fold or <0.6-fold) were considered as a significant up- or down-regulation, respectively (n = 3). C. Western blotting analysis illustrated increased expression levels of RTN3L, a tubular ER-phagy receptor, in TgR and TmR compared to HT22 WT. TUB was used as loading control. Values represent mean ± S.E.M.; n = 3, *p < 0.05, **p < 0.01 D-E. ER-phagy reporter ssRFP-GFP-KDEL was transiently expressed in HT22 cell lines and activated using doxycycline. The observed red puncta (GFP-/RFP + fragments) indicated an elevated in ER-phagic flux (n = 3, 22–25 transfected cells per group). Scale bars represent 10 μm. Immunoblots quantified the ratio of free RFP:RFP-GFP. Data represent the mean ± S.E.M., n = 4, ns non-significant, ∗p < 0.05; ∗∗p < 0.01. Arrowheads indicated RFP-positive puncta of the reporter. (For interpretation of the references to color in this figure legend, the reader is referred to the Web version of this article.)

Next, we investigated the expression level of 88 genes regulating different steps of autophagosome biogenesis and the non-canonical autophagy machinery via qPCR analysis. Overall, TgR demonstrated increased expression levels of genes involved in lipid transfer (*Atg9b*, *Wipi1*, *Wipi2*, *Wdr45l*) and LC3 lipidation (*Atg4b*, *Atg4d*, *Gabarap*, *Gabarapl2*) (Fig. 3B) in line with the observed increase in basal autophagic flux in TgR (Fig. 3A). Enhanced lipid supply might be required for the constant autophagic membrane turnover and repair caused by ER stress. Importantly, both ER stress-resistant cell lines showed increased expression of lysosomal marker *Lamp1*, cargo receptor *Sqstm1/p62*, Atg8 conjugation protein *Gabarapl2*, and lipid transport proteins *Atg9b*, *Wipi1*. The expression data confirmed that adaptation to chronic calcium stress causes massive rearrangements in autophagy.

ER-phagy, the selective autophagy pathway for ER turnover, is a critical player in organelle homeostasis [7,42]. During starvation, damaged or fragmented ER portions are selectively sequestered into autophagosomes via specific ER-phagy receptors, and subsequently degraded in (autophago)lysosomes [43]. To investigate ER-phagy, we determined the expression of ER-phagy receptors such as long isoform of reticulon-3 (RTN3L), atlastin-3 (ATL3), reticulophagy regulators (FAM134s), testis-expressed protein 264 homolog (TEX264), cell cycle progression protein 1 (CCPG1), and translocation protein (SEC62) [7,44]. Both ER stress-resistant cell lines showed up to 2.5-fold increased RTN3L levels compared with WT cells (Fig. 3C). ATL3 levels were 0.8-fold decreased in TgR cells, while FAM134s, TEX264, CCPG1, and SEC62 expression were unchanged (Figs. S3C and D). Surprisingly, we found no differences in CCPG1 and SEC62 that mediated ER-phagy during ER stress and recovery in other cell models [45,46]. To functionally monitor ER-phagy, we expressed an ER-phagy reporter construct (ssRFP-GFP-KDEL) as previously described [32]. Under basal conditions, WT cells exhibited an overall lack of ER-phagy (GFP-RFP co-positive puncta), while starvation conditions induced ER-phagy (RFP-only puncta) (Fig. 3D). In TgR and TmR, however, we detected RFP-only puncta already under nutrient-rich baseline conditions and following starvation (Fig. 3D). Next, we quantified ER-phagic flux (free RFP/GFP-RFP ratio), detecting the same reporter using immunoblotting. Consistent with our immunocytochemical analyses, we confirmed the previously observed starvation-induced increase in ER-phagy in WT cells. As expected, TgR and TmR showed an increased ratio already under nutrient-rich conditions (Fig. 3E). Moreover, RTN3L was detected only in lysosomal fractions of ER stress-resistant cells (Fig. S3E), suggesting that the ER-phagy receptor RTN3L is involved in the observed enhanced ER-phagy following prolonged ER stress. Taken together, chronic ER stress caused an enhanced ER-phagy response, as seen in the adapted cells.

### 3.4 Proteome analysis highlights changes in proteins associated with mitochondrial and metabolic functions in ER stress-resistant cells

Proteome analysis was performed using the nanoLC-ESI-MS/MS system as previously described [35,36]. Label-free quantification analysis of designated samples identified 1578 proteins with a false discovery rate (FDR) of 1 % (Supplementary data 05). In order to reveal differentially expressed proteins (Student's T-test, p-value <0.05), we used protein label-free quantitation (LFQ) intensity values of identified proteins extracted from MaxQuant analysis for statistical analysis utilizing the Perseus software. Among the identified proteins, we found 376 to be significantly differentially abundant among designated groups (Supplementary data S5). The hierarchical clustering of these proteins illustrated distinct expression patterns among WT, TgR, and TmR cells (Fig. 4A). Compared with WT, TgR and TmR displayed over 80 up- and over 100 down-regulated proteins. We further subjected the identified differentially abundant proteins to bioinformatics analysis and clustered them according to significantly regulated canonical pathways. In both resistant lines, the protein clusters were significantly associated with the cholesterol biosynthesis superpathway, the sirtuin signaling pathway, mitochondrial dysfunction, and oxidative phosphorylation (OXPHOS) (Fig. 4B). Interestingly, while we detected regulated proteins implicated in the UPR in both cell lines, only TgR showed regulated autophagy-related proteins (Fig. 4B). ER stress contributes to mitochondrial dysfunction and exacerbates fundamental metabolic processes [47]. Searching against genes encoding for mitochondrial proteins in the MitoCarta 3.0 collection [48], we discovered altered levels of 53 mitochondrial proteins in ER stress-resistant cells compared with HT22 WT. These proteins are involved in carbohydrate metabolism and OXPHOS input (some subunits of complex I and II). All of them display a reduced expression in both ER stress-resistant cells (Fig. 4C). The protein-protein interaction network analysis of these proteins mapped to the STRING database unveiled a functional cluster associated with the tricarboxylic acid cycle and OXPHOS (Fig. 4D). Taken together, the observed differential protein expressions patterns suggested that, in response to chronic ER stress, the resistant cell lines adaptively regulated a wide range of pro-survival pathways.

**Fig. 4:**
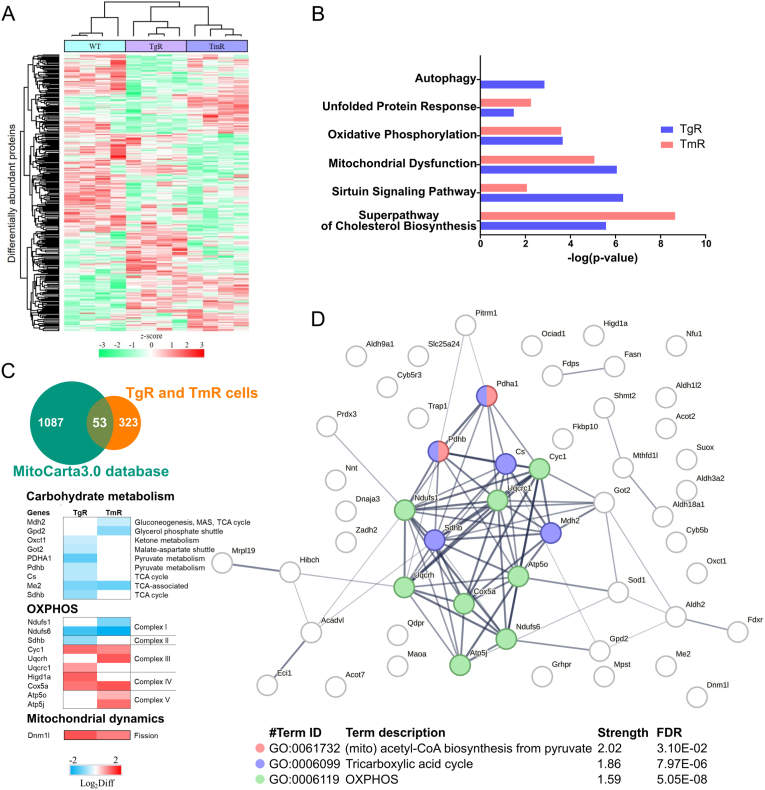
Proteome-wide analysis of HT22 WT, TgR and TmR cells A. The hierarchical clustering of the 376 differentially abundant proteins in the designated samples displayed in a heat map. The higher abundant proteins are shown in red and the lower abundant proteins are in green, with z-score of protein intensity displayed in color, n = 4. B. The top significant canonical pathways of the differentially expressed proteins are ranked by negative log10-transformed p-values in TgR and TmR cells C. The Venn diagram illustrates the 53 differentially abundant proteins in the resistant cells overlapped with MitoCarta 3.0. Heatmap of these proteins showed the changes in protein expression levels related to carbohydrate metabolism, OXPHOS, and mitochondrial dynamics. The data represent the z-scores of the differential expression (log2 scale). MAS malate-aspartate shuttle, TCA tricarboxylic acid. D. Protein–protein interaction network by STRING shows a cluster of proteins involved in biological processes of mitochondrial acetyl-CoA biosynthesis process from pyruvate, tricarboxylic acid cycle, and OXPHOS. The nodes indicate proteins, and edges indicate the strength of interactions. The thickness of the edges indicates the confidence score of network. (For interpretation of the references to color in this figure legend, the reader is referred to the Web version of this article.)

### 3.5 Metabolic reprogramming in response to chronic ER stress

**Fig. 5:**
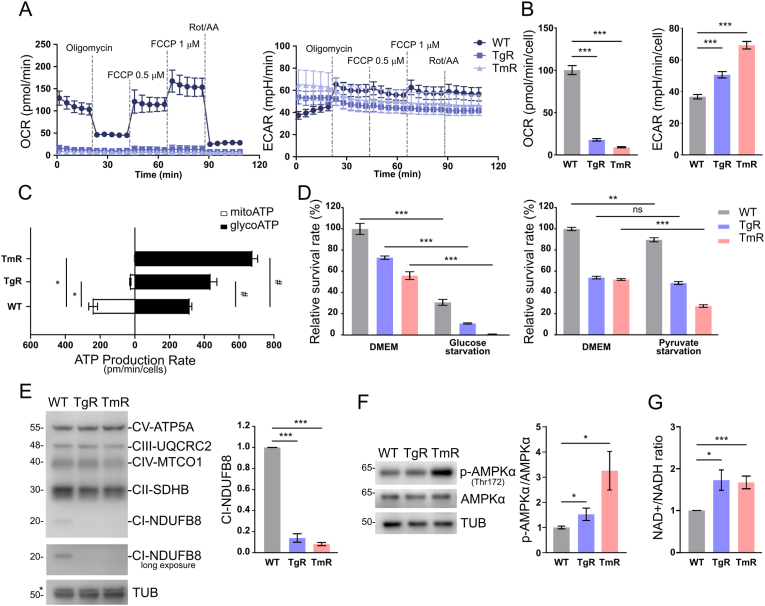
The shift to Warburg effect under prolonged ER stress A. Oxygen Consumption Rate (OCR) and Extracellular Acidification Rate (ECAR) profile plots in WT, TgR, and TmR cells with sequential injections of oligomycin (complex V inhibitor), FCCP (uncoupler), and a combination of rotenone and antimycin A (complex I and III inhibitor, respectively). B. OCR (pmol/min/cell) and ECAR (mpH/min/cell) under basal conditions. Values represent mean ± S.E.M., n = 21–32, ***p < 0.001 C. ATP Production Rates showed a metabolic switch towards aerobic glycolysis in the stress-resistant HT22 cells (decrease in mitoATP Production Rate and increase in glycoATP Production Rate) without significant changes in total ATP Production Rate. Data shown are mean ± S.E.M., n = 16, ns non-significant, *p < 0.05 vs. mitoATP group of HT22 WT, #p < 0.05 vs. glycoATP group of HT22 WT D. Cell viability of cells in glucose- or pyruvate-starved media for 48 h. Values represent mean ± S.E.M., n = 4, ***p < 0.001. E. Western blot analysis of OXPHOS complex subunits defined a reduction in expression of NDUFB8, a complex I subunit in TgR, and TmR compared to HT22 WT. TUB as loading controls. Values represent mean ± S.E.M., n = 3, ***p < 0.001. F. Immunoblots shows an upregulation of AMPK phosphorylation (Thr172) in the resistant cells. Values represent mean ± S.E.M., n = 4, *p < 0.05 G. The NAD+/NADH ratio of HT22 WT, TgR, and TmR cells under basal conditions. Mean ± S.E.M., n = 4, *p < 0.05, ***p < 0.001.

Ultrastructural analyses and proteomics depicted functional changes in mitochondria following chronic ER stress (Fig. 4B, Fig. S1Bg). Here, we monitored the real-time oxygen consumption rate (OCR) and extracellular acidification rate (ECAR) of cells using the Mito Stress Test. We found mitochondrial respiration to be completely abolished in TgR and TmR when compared with a normal respiration in WT controls (Fig. 5A). While adding mitochondrial uncoupler FCCP induced respiration in control cells, it was not inducible in resistant cells. Quantitation illustrated a massive decline of the basal OCR in stress-resistant cells compared to the WT. Correspondingly, stress-resistant cells display a surge in basal ECAR (Fig. 5B). In HT22 WT, the real-time ATP production rate showed about 56 % of the ATP resulted from glycolysis with the other short half deriving from mitochondrial OXPHOS. While TgR cells mostly produced ATP through glycolysis, TmR derived 100 % of their ATP production from glycolysis (Fig. 5C). These findings imply that, under prolonged ER stress, cells undergo a significant metabolic shift from mitochondrial respiration to aerobic glycolysis, also known as ‘Warburg-like effect’.

Next, we challenged WT, TgR, and TmR with either glucose- or sodium pyruvate-deprived conditions for 48 h. Glucose starvation induced more intense cell death in TgR or TmR than in WT cells, while pyruvate starvation showed only minor alterations (Fig. 5D). This suggests that aerobic glycolysis could play a significant role in chronic ER stress survival. Due to the altered expression of proteins of the OXPHOS machinery observed in the proteomics analysis (Fig. 4C), we investigated the protein levels of some respiratory complex I–V subunits using an OXPHOS antibody cocktail. Only NADH dehydrogenase [ubiquinone] 1 beta subcomplex subunit 8 (NDUFB8) level, a CI subunit, was reduced by ∼ 10 % compared to parental HT22 WT cells, while other subunits appeared unchanged (Fig. 5E). AMP-activated protein kinase (AMPK), an energy sensor, enhances ATP production by increasing glucose uptake and glycolysis [49,50]. Indeed, phosphorylation of AMPKα at Thr172 was stimulated in TgR and TmR (Fig. 5F). AMPK induces cellular nicotinamide adenine dinucleotide (NAD+) levels that regulate energy metabolism [51] and autophagy-controlled NAD levels are crucial for cell survival [52]. Upon aerobic glycolysis, cells regenerate NAD+ from NADH by reduction of pyruvate to lactate. Consistently, the observed increase in glycolysis in TgR and TmR is accompanied with an increase in the NAD + -to-NADH ratio evidenced via a fluorescence-based assay (Fig. 5G). In fact, complex I oxidizes NADH to NAD + when cells respire. The observed reduction in complex I subunits (Fig. 4, Fig. 5E), along with the increased NAD + -to-NADH ratio (Fig. 5G), argue for regenerating NAD + via lactate production in ER stress-resistant cells and is consistent with the observed activation of aerobic glycolysis. Taken together, these results suggest an adaptive role of the Warburg-like effect in enduring long-term ER stress in TgR and TmR cells.

### 3.6 ER stress-resistant cells divergently regulate the UPR

**Fig. 6:**
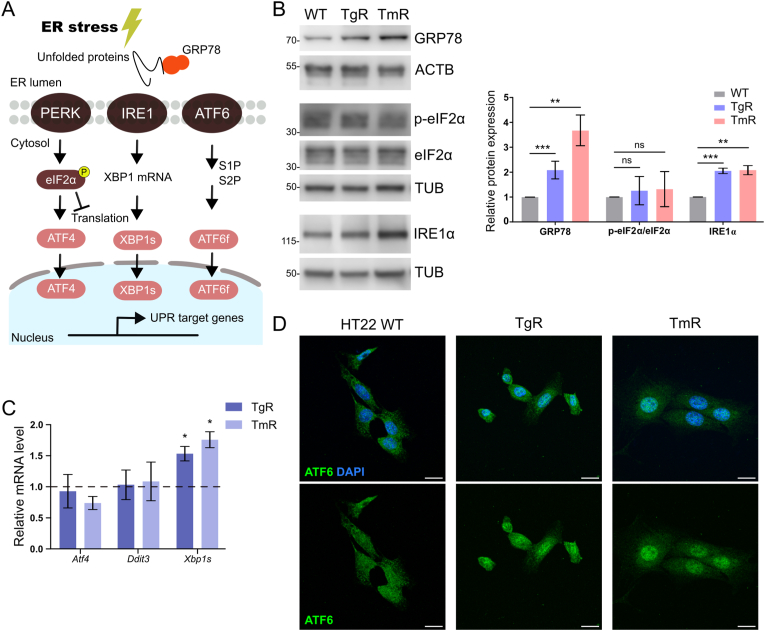
Divergent upregulation of the UPR in ER stress-resistant cells A. Schematic representation of the UPR pathway B. Immunoblots illustrated the elevated expression of UPR components in chronic ER stress. Actin or Tubulin was used as loading control. Values represent mean ± S.E.M., GRP78: n = 4, IRE1α and *p*-eIF2/eIF2α: n = 3, ns non-significant, **p < 0.01, ***p < 0.001 C. qPCR results indicated the relative expression level of *Xbp1s*, *Atf4* and *Ddit3* mRNA in TgR and TmR cells. GAPDH was used as control. Data are represented as mean ± S.E.M., *Xbp1s*: n = 4, *Atf4* and *Ddit3*: n = 3, *p < 0.05. The control expression of HT22 WT cells was set to 1 D. Immunofluorescence labeling showed the translocation of ATF6 into nucleus. DAPI (blue) was used to stain DNA. Scale bars: 20 μm. n = 3, 80–100 cells each group. (For interpretation of the references to color in this figure legend, the reader is referred to the Web version of this article.)

In an adaptive state, the UPR is activated to maintain proteostasis and impacts various cell survival responses (Fig. 6A). We detected induction of IRE1α expression and *Xbp1s* mRNA expression in both ER stress-resistant cells compared with controls (Fig. 6B and C). Moreover, TgR and TmR cells both demonstrated enhanced nuclear translocation of ATF6 (Fig. 6D) and elevated expression of GRP78, a signal for ATF6 induction (Fig. 6B). Notably, GRP78 is also critical for the control of the ER structure and stress-induced autophagosome formation [53]. Interestingly, we observed no increase in eIF2α phosphorylation under chronic ER stress, while WT cells displayed significantly elevated protein levels of GRP78, IRE1α, and *p*-eIF2α during acute stress (Figs. S4A and B). Consistently, *Atf4* and *Ddit3* mRNA levels in either TgR or TmR remained unchanged (Fig. 6C). IRE1 and ATF6 branches support cell survival in human ER-stressed cells, however, they are attenuated by pro-apoptotic ER stress [54]. The differential control of three UPR arms indicate a selective response based on the particular stress applied, which could be important for resistance development.

## 4 Discussion

Aberrant cellular energetics, disturbed proteostasis, and homeostasis of organelles, including the ER, are associated with neurodegenerative diseases [55]. Exposing to amyloid-β enhances cytosolic calcium levels as well as vulnerability to excitotoxicity in neurons [56]. Recent N-glycoproteome analyses of cerebrospinal fluid from AD patients demonstrated global changes in the expression pattern of glycoforms [57]. The dysregulation of Ca^2+^ signaling as well as N-linked glycosylation observed in AD affects ER functions and leads to ER stress.

Alzheimer-associated neurodegeneration damages neurons unequally; selective brain areas including the entorhinal cortex, hippocampus, temporal cortex, frontal cortex, cingulate cortex, amygdala, and nucleus basalis of Meynert are more vulnerable to AD [58]. Interestingly, three brain regions including temporal cortex, frontal cortex, and hippocampus show increase in levels of BiP, phosphorylated PERK, IRE1, and ATF6 in response to Tm injection but not the cerebellum, a resistant brain region to AD [59]. Moreover, susceptibility to Tm is different among hippocampal subregions and is ranked dentate gyrus > CA1 > CA3 [60].

Here, we thoroughly analyzed cell signaling pathways in response and adaptation to chronic ER stress provoked by Tg or Tm in hippocampal neuronal cells. Calcium dysregulation-mediated ER stress transcriptionally upregulated genes related to lipid transport and the autophagy-associated lipidation machinery, which may allow increased AV formation. Both TgR and TmR cells showed an enhanced ER-phagic activity under basal conditions (Fig. 3), suggesting that cells maintained a new steady-state ER phagy level despite challenges posed by accumulation of unfolded proteins. In addition, changes in expression of RTN3L and ATL3 imply that an effective turnover of ER tubules and tubule junctions plays an important role in the adaptation to chronic ER stress. Indeed, RTN3L drives degradation of protein aggregates in the ER lumen [[61], [62], [63]].

Interestingly, the bioenergetics changes including decreased mitochondrial respiration and Warburg-like metabolic reprogramming (Fig. 5) indicate a compensatory metabolic response of neuronal cells during ER stress to meet energy demands [64]. In fact, short-term ER stress promotes respiratory supercomplexes and enhances OXPHOS [65]. An altered glucose metabolism is one of the hallmarks of cancer cells but also exists in non-malignant cells [66]. Warburg-like metabolism as an adaptive response was reported to protect neurons during early stages of AD, but worsens pathophysiology during late stages of AD [67]. Metabolic reprogramming to aerobic glycolysis in neurons seen in AD was interpreted as a measure to survive and cope with neurodegenerative challenges [68,69]. Under physiological conditions, neurons are mainly oxidative while glial cells including astrocytes and oligodendrocytes promote glycolytic metabolism [70,71]. Transferring lactate from astrocytes to neurons is responsible for long-term potentiation and memory consolidation [72] and extracellular lactate modulates neuronal excitability [73]. Furthermore, lactate was shown to display neuroprotective effects against glutamate-induced excitotoxicity, traumatic brain injury, and psychiatric disorders [72]. These reports highlight neuroprotective and signaling roles of lactate in addition to its key role in energy metabolism, suggesting that the activation of aerobic glycolysis may provide lactate for energy needs as well as a protective response upon chronic ER stress.

The activation of IRE1 and ATF6 pathways, but not PERK (Fig. 6), implies that the severity and duration of ER stress demonstrates a differential involvement of UPR. The simultaneous activity of IRE1 and ATF6 pathways may promote the adaptive UPR and prevent apoptosis under chronic ER stress. In fact, sustained IRE1 signaling supports cell proliferation in the face of chronic ER stress while equivalent conditions of selective PERK activation demonstrate impaired cell proliferation and elevated cell death [74]. Embryonic fibroblasts and mice with ATF6α deletion show disruption in ER protein processing and fail to adapt to chronic ER stress [75]. Artificial heterodimers of XBP1s and ATF6f ameliorate the clearance of aberrant aggregation *in vivo* [76]. Further, IRE1α promotes RTN3L-mediated ER-phagy in podocytes during Tm-provoked ER stress [77], suggesting a link between the UPR and ER-phagy in response to chronic ER stress.

We observed many changes in TgR and TmR cell morphology and organelle architecture employing microscopy (TEM and confocal). In addition to increased numbers of AVs, we observed very large (‘giant’) lysosomes (Fig. 1, Fig. 2), suggesting that chronic ER stress drives the formation and accumulation of AVs. An increase in ER stress is a hallmark of aging [78]. Consequently, effectively coping with ER stress could be one additional measure to prevent age-related neurodegeneration as caused by alterations in autophagic-lysosomal activity. However, the activity of cathepsins is insufficient for an effective lysosomal degradation under the applied ER stress in both adapted cell lines.

In summary, here we show the adaptability of clonal neuronal cells to chronic ER stress induced by dysregulation of calcium signaling or glycosylation. TgR, but not TmR cells, displayed enhanced general autophagy independent of mTOR activity. Phosphorylation of ULK1, lipid transport proteins, and the lipidation machinery, as well as critical proteins for autophagy initiation and autophagosome elongation, were increased under persistent ER stress induced by calcium perturbation. Intriguingly, in both resistant cell lines, ER-phagy culminated in high expression levels of RTN3, a receptor of ER tubule degradation. Our MS-based proteomics analysis uncovered the differential expression of numerous proteins suggesting a diverse set of key pathways including mitochondrial dysfunction and OXPHOS to be involved in ER stress adaptation. In addition to the observed Warburg-like metabolic pattern in ER stress-resistant cells, the selective activation of IRE1 and ATF6 signaling pathways highlights the distinct roles of three UPR transducers in acute and chronic stress. Taken together, these findings demonstrate a high plasticity and adaptation capacity of neuronal cells to long-term ER stress *in vitro* and may pave the way to find regulators of this ER stress adaption. Potentially, TgR and TmR cells could be effective tools to screen for compounds that may on one hand revert the resistance phenotype and on the other hand selectively activate a molecular response that provides resistance against chronic ER stress.

## Author contribution

CB &JS conceptualized the project; TNMP planned and performed the central experiments; NP & CM provided the proteomics/bioinformatics; SE & MB performed EM; DCF provided the metabolomics data; CUP, AMC & HK provided key input. TNMP & CB wrote the manuscript.

## Funding

This study was supported by grants of the CRC1177 of the 10.13039/501100001659Deutsche Forschungsgemeinschaft (DFG) to Christian Behl and Stefan Eimer, the 10.13039/501100001655Deutscher Akademischer Austauschdienst (DAAD) to Thu Nguyen Minh Pham. Natarajan Perumal, Caroline Manicam, and Dominik C. Fuhrmann are supported by grants from 10.13039/501100001659DFG (PE 2531/4–1 and MA 8006/1-1, SPP2306, respectively).

## Declaration of competing interest

There is no conflict of interest of all authors regarding the manuscript “Adaptive responses of neuronal cells to chronic endoplasmic reticulum (ER) stress”.

## Figures and Tables

**figs1:**
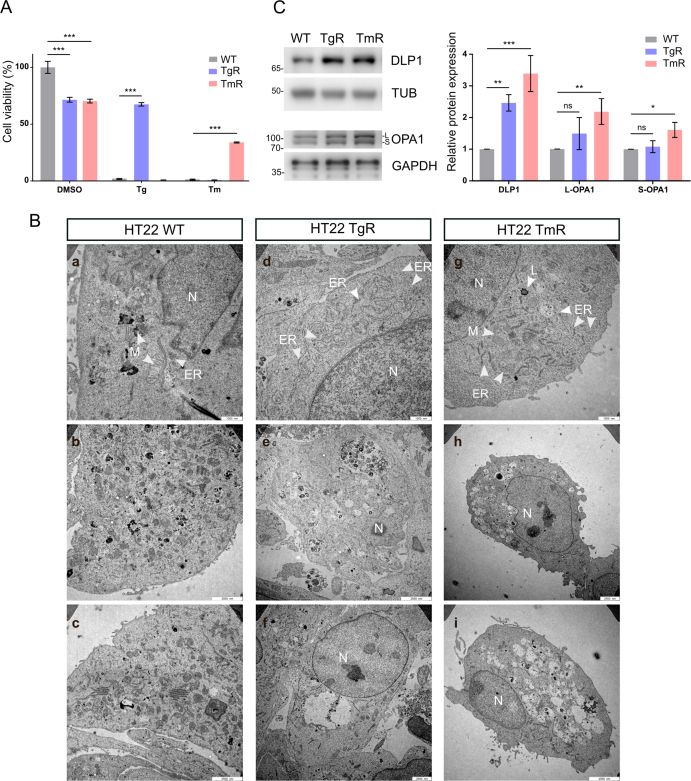


**figs2:**
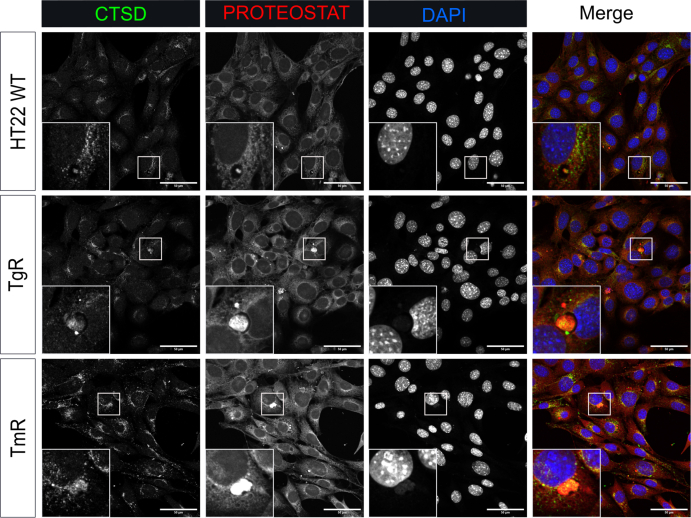


**figs3:**
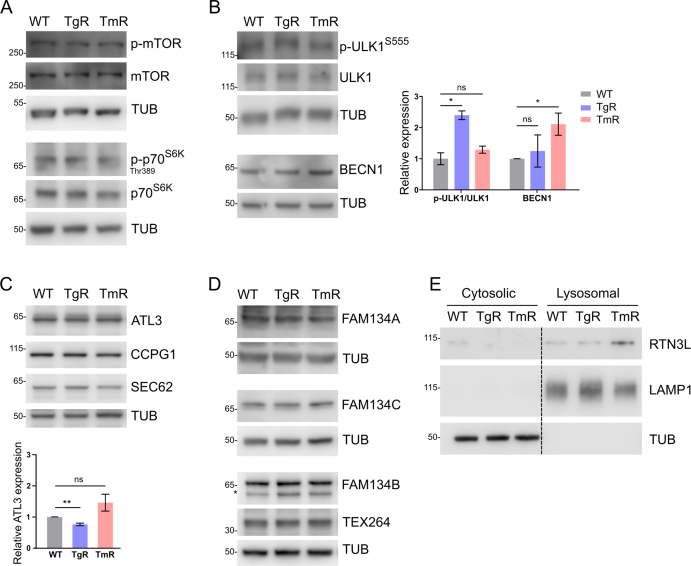


**figs4:**
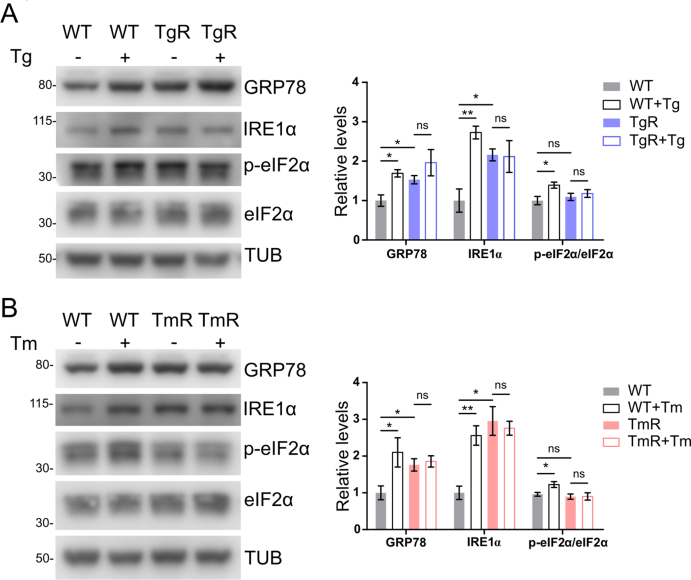


## Data Availability

Data will be made available on request.
