# Supplementary material for: Adaptive responses of neuronal cells to chronic endoplasmic reticulum (ER) stress
Source: Redox Biol. 2023 Oct 20;67:102943. doi: 10.1016/j.redox.2023.102943 (PMC10618786; doi:10.1016/j.redox.2023.102943)
Supplement: Multimedia component 1 [file mmc1.docx]

Figure Sup.1. Supplements for cell survival, ultrastructural analysis, and mitochondrial dynamics markers

A. Proliferation rate of HT22 WT, TgR, and TmR cells incubated in Tg 1.5 μM and Tm 1.5 μg/ml was measured by MTT assay over 48 h. No cross-resistance was detected in two ER stress-resistant cell lines. Values represent mean ± S.E.M., n = 4, *** p<0.001.

B. Ultrastructure analysis showed changes in the morphology of organelles of TgR and TmR compared to HT22 WT cells. Lysosomes (L), endoplasmic reticulum (ER), mitochondria (M), and nucleus (N). Scale bar: a, d, g 1000 nm; b, c, e, f, h, i 2500 nm.

C. Immunoblot analysis of DLP1 and OPA1 in HT22 WT, TgR, and TmR cells. GAPDH or Tubulin was used as loading control. Values represent mean ± S.E.M., DLP1: n = 3, OPA1: n = 4, ns non-significant, *p < 0.05, **p < 0.01, ***p < 0.001.

**Figure Sup.2. Misfolded/ aggregated proteins colocalize with CTSD in chronic ER stress**

Fluorescence images showed misfolded/ aggregated proteins compacted in enlarged structures that recruited CTSD in TgR and TmR cells. DAPI (blue) was used to stain DNA. Scale bars: 50 μm. Alexa Fluor^TM^ 647 was used in CTSD detection, the LUT was changed to green.

Figure Sup.3. Expression of autophagic regulators and ER-phagy receptors in cells.

A-D. Western blot analysis for major regulators of canonical autophagy (A, B) and ER-phagy receptors (C, D). The diagrams displayed the indicated protein levels normalized to TUB. This showed increases in phosphorylated ULK1 in TgR and BECN1 in TmR cells. Values represent mean ± S.E.M., p-ULK1/ULK1 and ATL3: n = 3, BECN1: n = 5, ns non-significant, *p < 0.05, **p< 0.01. Control HT22-WT cells were set to 1.

E. Translocation of RTN3L in TgR and TmR. The cells were fractionated into cytosolic and lysosomal fractions. Tubulin and LAMP1 were used as control for cytosolic and lysosomal fractions, respectively.

Figure Sup.4. Different responses of the UPR pathways in acute and chronic ER stress

A, B. Western blot analysis of the UPR pathways with/without the treatment of Tg 1.5 μM or Tm 1.5 μg/ml. The diagrams display the protein levels after normalization to tubulin. Values represent mean ± S.E.M., GRP78 and IRE1α: n = 3, p-eIF2α/ eIF2α: n = 4, ns non-significant *p < 0.05, **p < 0.01. Control HT22-WT cells were set to 1.
